# Reductions in Labor Capacity from Intensified Heat Stress in China under Future Climate Change

**DOI:** 10.3390/ijerph17041278

**Published:** 2020-02-17

**Authors:** Xingcai Liu

**Affiliations:** Key Laboratory of Water Cycle and Related Land Surface Processes, Institute of Geographical Sciences and Natural Resources Research, Chinese Academy of Sciences, Beijing 100101, China; xingcai@igsnrr.ac.cn

**Keywords:** wet-bulb globe temperature, humidity, heat stress, labor capacity, climate change

## Abstract

Heat stress would be intensified under global warming and become a key issue of occupational health for labor force working outdoors. The changes in labor force would affect regional socioeconomic development. So far, changes in labor force due to heat stress are not well documented in China. In this study, heat stress based on wet-bulb globe temperature (WBGT), which combines the thermal effects on the human body of both temperature and humidity, is projected for the near future (2021–2050) and the end of the century (2071–2099). Changes in labor capacity are then estimated for heavy and light work based on the relationships between labor capacity and the WBGT. Low and high emission scenarios, namely Representative Concentration Pathway (RCP) 2.6 and RCP8.5, are considered for the future projections in the hottest two months (July and August) in China. Results suggest that the WBGT would increase by more than 3–5 °C by the end of the century. The labor capacity would decrease by more than 40% for both heavy and light work in considerable areas such as South and East China, where there is a large population and developed economy. This indicates that labor force would reduce significantly due to intensified heat stress. This study calls for special attention to the impact of heat stress on occupational health and the labor force in China in the future.

## 1. Introduction

Heat stress often brings thermal discomfort or heat injury to human bodies when people are exposed to ambient air [1]. Heat stress has significantly increased during the past decades [2,3,4] and will be intensified in many regions of the world in the future [5,6]. Heat stress is largely determined by surface air temperature and thus is often projected to be more severe and more frequent under global warming [7]. Severe heat stress due to high temperature may lead to high mortality during the summer in China [8,9,10]. On the other hand, high humidity combined with a high temperature can further elevate heat stress levels that make hot weather more oppressive [11]. The combination of high temperature and high humidity has resulted in considerable death in South Asia [12]. 

Heat stress has been projected to increase in the future [13] due to global warming and elevated humidity [14], which would narrow the adaptability of humans to climate change [15]. The body temperature of humans is around 37 °C, while skin temperature is about 35 °C or lower under normal conditions for conducting metabolic heat [15]. Thus, avoiding exposure to an ambient temperature above 35 °C can reduce the risk of hyperthermia, because human skins have to maintain high evaporation to manage body temperature. The heat stress indicated by the wet-bulb globe temperature (WBGT), a heat stress index used for outdoor sports and training [16], has typically been low (below 31 °C) throughout most historical periods [15]. Under the Representative Concentration Pathway (RCP) 8.5 scenario, the air temperature would increase more than 6 °C in considerable regions of China, which would result in much more oppressive weather leading to a WBGT that is often larger than 35 °C [7]. Therefore, the changes in heat stress caused by global warming would have a significant impact on humans.

Due to global warming and population growth, about 48% of the global population is projected to be exposed to the climatic conditions exceeding the lethal heat threshold [17]. This would be particularly pronounced in urban areas. For example, in the city of Chicago, about 166 to 2217 excess deaths per year are projected under different scenarios for the 2081–2100 period. In the North China Plain, which is largely covered by rural areas, the risk from deadly heatwaves would increase under the business-as-usual scenario [18]. The WBGT extremes have been projected to approach and exceed the critical threshold (35 °C) in the region around the Arabian Gulf, which would lead to possible premature death [19]. Therefore, future changes in heat stress and impact on public health will be a major issue with respect to the adaptation to future climate change.

Exposure to heat stress will reduce productive working time [20], and may also decrease labor force participation due to weaker health. Elevated heat stress would cause work productivity loss by as much as one month at 2 °C global warming in developing countries [21]. In Beijing, the work time will decrease by 0.57% with a WBGT increase of 1 °C [22]. It may take a very high cost to mitigate the changes in work productivity resulting from climate change. The cost caused by heat-related reduction in work performance is about USD 655 per person annually in Australia [23], and in China it was estimated at USD 6.22 billion in total annually [24]. Many studies have been conducted to examine the impact of climate change on labor capacity/productivity at global and regional scales [25,26]. However, the standards for industrial and occupational work are often different across countries and regions [27]. Therefore, more local analysis is needed to demonstrate the potential impact of climate change. The impact of heat stress on the labor capacity in some cities in China has been reported (e.g., [20,22]), but the impact of increasing heat stress on outdoor work productivity over China on the whole under global warming is not well documented [28]. It has been suggested that increasing heat stress will be a key occupational health problem, and thus reduce the labor capacity globally [29]. In this study, we aim to investigate the change in heat stress indicated by the WBGT and estimate the potential impacts on the labor capacity of China in the future. This study could be a preliminary reference for assessing the impacts of climate change on occupational health and the associated adaptation of humans to its impacts.

## 2. Materials and Methods 

### 2.1. Climatic Data

Heat stress is estimated based on the climate projections from five generic circulation models (GCMs), namely GFDL-ESM2M, HadGEM2-ES, IPSLCM5A-LR, MIROC-ESM-CHEM, and NorESM1-M, which are provided by the Inter-Sectoral Impact Model Intercomparison Project (ISIMIP, https://www.isimip.org/). Daily average, maximum, and minimum temperature and specific humidity data over the period 1981–2099 are used in this study. These data were spatially interpolated and bias-corrected using an observation-based dataset. Water and Global Change (WATCH) forcing data [30] were used to obtain projections with a finer spatial resolution of half a degree [31]. These data have been widely used for global assessment of climate change impact (e.g., [32,33,34]), and have been validated with observations for heat stress assessment in China [7]. Two Representative Concentration Pathways (RCPs)—the low mitigation scenario (RCP2.6) and the high baseline emission scenario (RCP8.5)—are considered in this study for representing the scenarios of climate change.

### 2.2. Wet-Bulb Globe Temperature (WBGT)

The WBGT was initially used by the United States Army Marine Corps to monitor ambient conditions and reduce thermal injuries during outdoor training and campaigns [35]. So far, it has been widely used as a heat stress index, accounting for both temperature and humidity [16]. The simplified WBGT is used as a proxy of potential heat stress as follows:

WBGT = 0.567*T* + 0.393*e_a_* + 3.94, (1)

where *T* (°C) is daily temperature and *e_a_* (hPa) is water vapor pressure. Equation (1) was established under moderately high radiation and light wind conditions; therefore, it may slightly overestimate heat stress on cloudy or windy days and underestimate the days with full sun and small winds [36].

### 2.3. Labor Capacity

**Figure 1:**
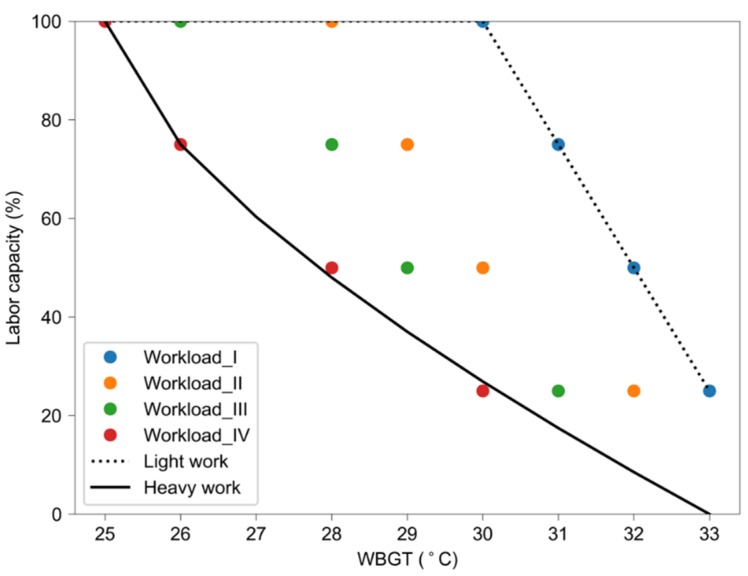
Labor capacity at different threshold limit values of the WBGT. The black line indicates the fitted relationship between labor capacity and the threshold limit values for heavy works (Workload IV), while the black dashed line indicates this relationship for light works (Workload I). See Table 1 for related numbers.

Labor capacity is defined as the occupational capacity (working time) to safely perform sustained labor under environmental heat stress [29]. Extreme environmental conditions (e.g., high temperature, solar radiation, wind, and snowfall, etc.) would reduce the capacity of the labor force working outdoors. The labor capacity would be 100% (8 hours per day) under normal conditions. In this study, the impact of heat stress on the labor capacity of heavy and light works is assessed by using empirical relationships between labor capacity and the WBGT based on the threshold limit values (Table 1) recommended by the Chinese standard [37]. To smoothly estimate the labor capacity under different heat stresses, the threshold limit values for heavy (workload IV in Table 1) and light (workload I in Table 1) works were fitted (Figure 1), respectively, by using the formulas that are the same or similar to Dunne et al. [29]. The empirical relationship for heavy work is estimated as

LC = 100 − 25 × max (0, WBGT-25)^2/3^, (2)

while for light work, the labor capacity is estimated by a linear relationship wherein the labor capacity reduces at a WBGT higher than 30 °C:

LC = 100 − 25 × max (0, WBGT-30).(3)

The labor capacity is not a direct physiological limit, but represents the capacity of a healthy, acclimated individual to safely perform heavy labor under environmental heat stress. It is estimated as a percentage of the standard labor (e.g., 8 h per day) under the conditions without any heat stress.

### 2.4. Spatial and Temporal Analysis

**Figure 2:**
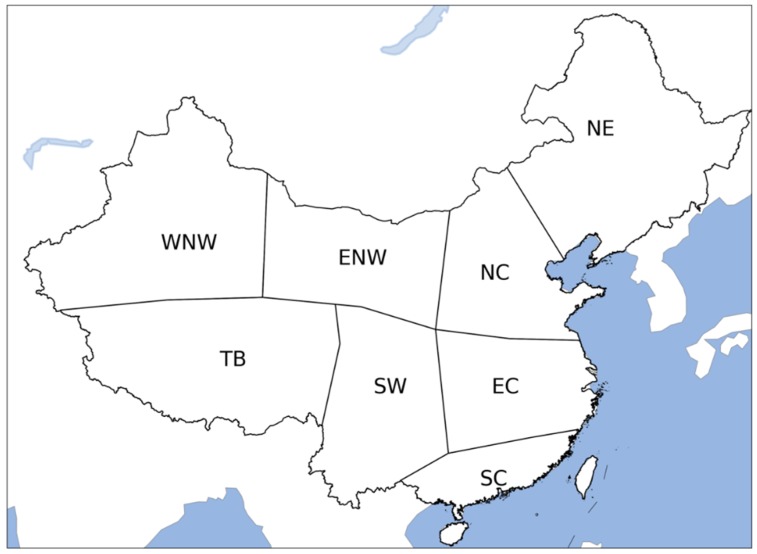
Climate regions of China’s land area used in this study. WNW: western Northwest China, ENW: eastern Northwest China, N: North China, NE: Northeast China, TB: Tibet, SW: Southwest China, E: East China, S: South China.

We performed this study in July and August, the two hottest months in China, at grid cell and regional scales. Eight regions were delineated for China’s land area, based mainly on their climate conditions (Figure 2). Spatial analysis was then conducted for grid cells and the eight regions. Time series of labor capacities were spatially aggregated over the eight regions for temporal analysis.

Multimodel ensemble medians were used for the main analysis. For individual combinations of RCPs and GCMs, the heat stress was estimated for three time periods: the baseline period (1981–2010), the near future (2021–2050), and the end of the century (2071–2099). The 25th and 75th percentiles were calculated for temporal analysis to show the multimodel uncertainties in the estimates of labor capacity.

## 3. Results

### 3.1. The Changes in the WBGT

**Figure 3:**
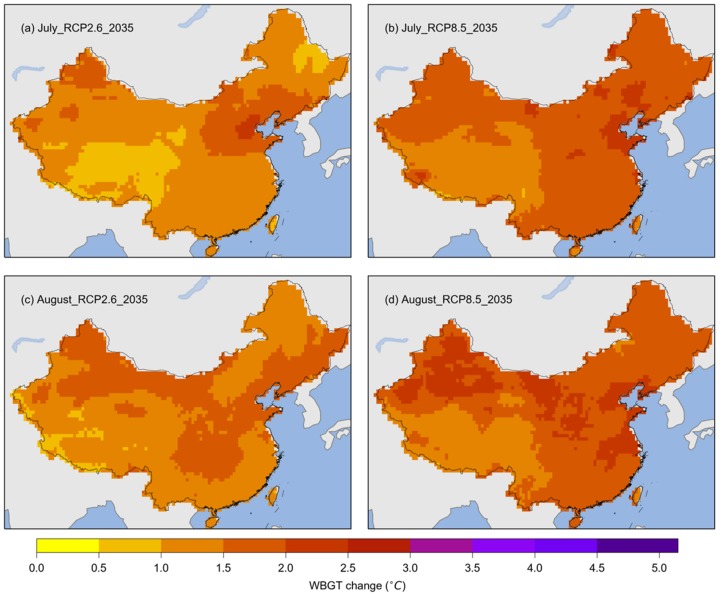
Multimodel ensemble medians of the changes in WBGT for July and August during the period 2021–2050 (labeled as 2035). (**a**) Multi-year means of the WBGT for July during the 2021–2050 period under RCP2.6; (**b**) multi-year means of the WBGT for July during the 2021–2050 period under RCP8.5; (**c**) multi-year means of the WBGT for August during the 2021–2050 period under RCP2.6; (**d**) multi-year means of the WBGT during August for the 2021–2050 period under RCP8.5.

Figure 3 shows the projected changes in WBGT for July and August in China for the near future (2021–2050) compared with the baseline period (1981–2010). For July, the WBGT is projected to increase over all of China. For both RCPs, the WBGT would increase by more than 1 °C in most areas. In many parts of the Tibetan Plateau, the WBGT would increase by less than 1 °C in July under RCP2.6, but by more than 1 °C under RCP8.5. An increase of more than 1.5 °C is projected for all of China according to the July WBGT under RCP8.5. For August, the WBGT would increase by more than 1 °C in almost all of China, with larger areas increasing by more than 1.5 °C. Considerable areas showing increases larger than 2 °C were found for the August WBGT under RCP8.5.

**Figure 4:**
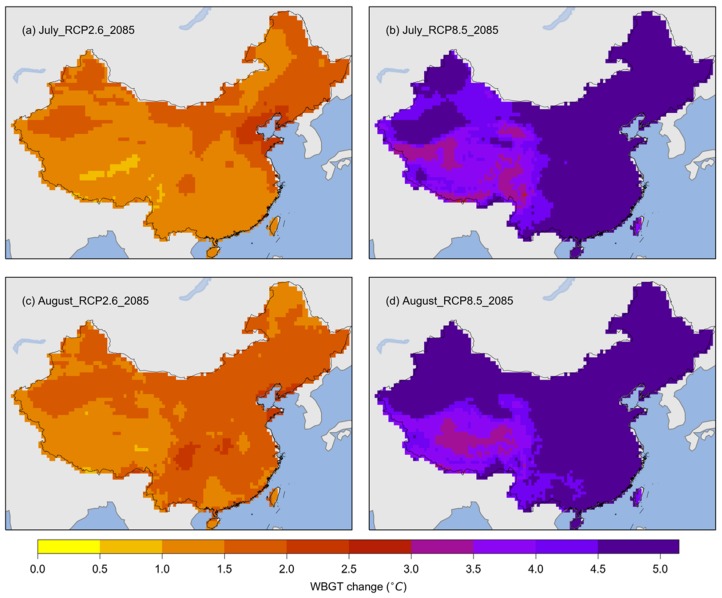
Multimodel ensemble medians of the changes in WBGT for July and August during the period 2071–2099 (labeled as 2085). (**a**) Multi-year means of the WBGT for July during the 2071–2099 period under RCP2.6; (**b**) multi-year means of the WBGT for July during the 2071–2099 period under RCP8.5; (**c**) multi-year means of the WBGT for August during the 2071–2099 period under RCP2.6; (**d**) multi-year means of the WBGT for August during the 2071–2099 period under RCP8.5.

Figure 4 shows the projected changes in the WBGT for July and August in China at the end of the century (2071–2099) compared with the baseline period (1981–2010). For the RCP2.6 scenario, more areas showed increases of larger than 1 °C, but a few areas showed increases of more than 1.5 °C. However, for the RCP8.5 scenario, larger increases in WBGT were found for both July and August. The WBGT would increase by more than 3 °C over all of China, and increases of more than 4.5 °C were found in many areas. 

### 3.2. The Spatial Patterns of the Future Labor Capacity

**Figure 5:**
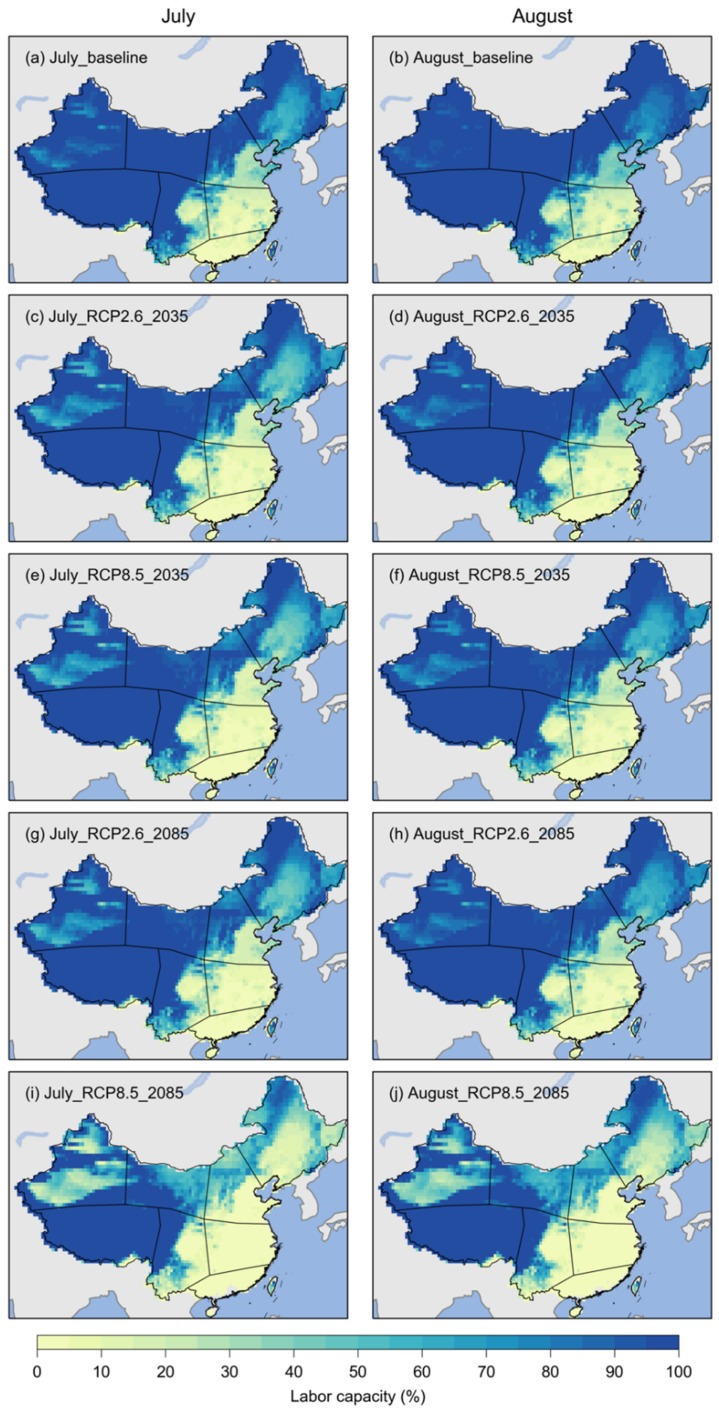
The labor capacity of heavy works in China in July and August for the baseline and 2021–2050 periods (labeled as 2035) under RCP2.6 and RCP8.5. (**a**) and (**b**) labor capacity for July and August, respectively, during the 1981–2010 period (baseline); (**c**) and (**d**) labor capacity for July and August during the 2021–2050 period under RCP2.6; (**e**) and (**f**) labor capacity for July and August during the 2021–2050 period under RCP8.5; (**g**) and (**h**) labor capacity for July and August during the 2071–2099 period under RCP2.6; (**i**) and (**j**) labor capacity for July and August during the 2071–2099 period under RCP8.5.

Figure 5 shows the projected labor capacity for heavy works during July and August in China for the baseline period (2021–2050) and the 2071–2099 period. The labor capacity has similar spatial patterns for the three periods for both July and August under RCP2.6 and RCP8.5 (i.e., low in Southeast China and high in western China). For the three periods, the labor capacity would be larger than 90% in the Tibetan Plateau and larger than 80% in most areas of northern China under the two RCPs. Labor capacity would be less than 10% in many areas of South China and East China. Compared with the baseline period, the labor capacity in the future, especially by the late 21st century, would be smaller in most areas except for the Tibetan Plateau. Future changes in labor capacity would be particularly significant in northern China under RCP8.5 (e.g., Figure 5i,j). The labor capacity for heavy works would be slightly larger in August than in July.

**Figure 6:**
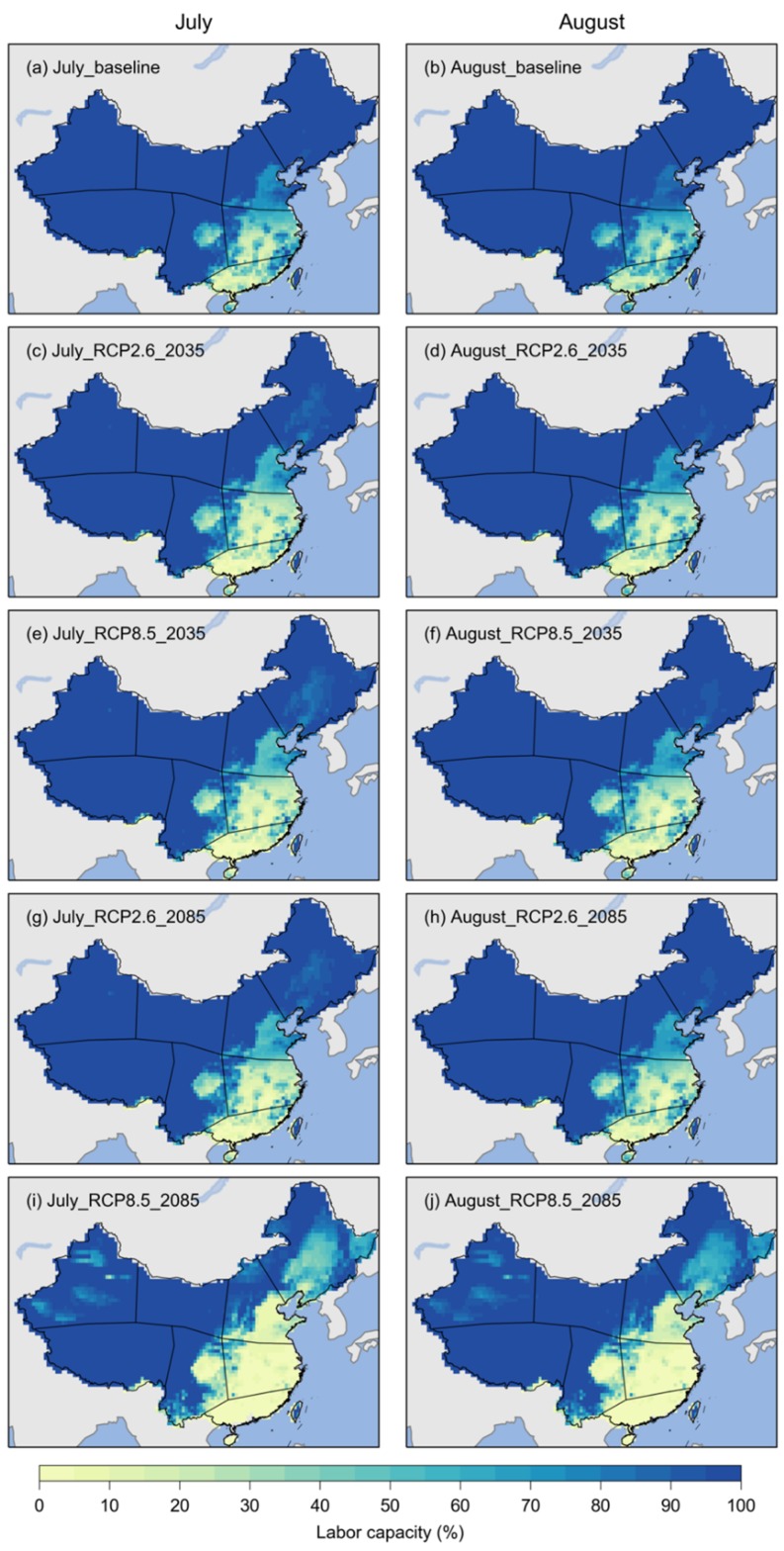
The labor capacity of light works in China for July and August during the 2071–2099 period (labeled as 2085) under RCP2.6 and RCP8.5. (**a**) and (**b**) labor capacity for July and August, respectively, during the 1981–2010 period (baseline); (**c**) and (**d**) labor capacity for July and August during the 2021–2050 period under RCP2.6; (**e**) and (**f**) labor capacity for July and August during the 2021–2050 period under RCP8.5; (**g**) and (**h**) labor capacity for July and August during the 2071–2099 period under RCP2.6; (**i**) and (**j**) labor capacity for July and August during the 2071–2099 period under RCP8.5.

Compared to the baseline period, the labor capacity of light works in July and August showed relatively small changes in many areas of China for the 2021–2050 period, but relatively large changes during the 2071–2099 period, particularly under RCP8.5 (Figure 6). Under RCP2.6, the labor capacity in the 2071–2099 period (Figure 6g,h) would be generally similar to that during the 2021–2050 period (Figure 6c,d), showing slightly less labor capacity than the baseline period. For RCP8.5, the labor capacity would still be larger than 90% of the Tibetan Plateau and surrounding areas, but less than 70% of considerable areas of northern China during the 2071–2099 period (Figure 6i,j). The labor capacity in the two months would be smaller than 10% in many areas of southeastern China for both RCPs.

The labor capacity of heavy work during the baseline period and the changes in the future are shown in Table 2. For the whole of China, the labor capacity overall is around 85% for July and August during the baseline period. Under RCP2.6, the labor capacity of China would decrease by about 4% to 4.6% in the future during July and August. That means the labor capacity would be at about 81% at that time. Under RCP8.5, the labor capacity of China in July (August) would decrease by 5.5% (5.6%) and 17% (16%) during the 2021–2050 and 2071–2099 periods, respectively. In other words, the labor capacity of China would be around 80% in the near future, and less than 70% in the late 21st century.

The largest labor capacity in the baseline period was found in Northwest China, and was more than 95% and 97% for July and August, respectively. The Tibetan Plateau had the next highest labor capacity, at more than 93% for the two months during the baseline period. The labor capacity in Northeast China was also high (about 85% for July and 91% for August), while it was often around 70% or less in North China and Southwest China. The labor capacity was smallest in South China, at only 6% and 10% in July and August, respectively, and it was less than 25% for July and August in East China.

The changes in labor capacity generally ranged from −0.8% to −13% in the 2021–2050 period to more than −40% during the 2071–2099 period. In almost all regions, the changes under RCP2.6 were relatively smaller than those under RCP8.5. Generally, the changes in July were larger than those in August in northern China (e.g., Northeast, Northwest, and North China). However, a contrast was found in the Tibetan Plateau and southern China (e.g., South China, East China, and Southwest China). The smallest changes in labor capacity were found in the Tibetan Plateau, which could decrease by around 1% under RCP2.6 or in the near future under RCP8.5, and decrease by about 4% in the late 21st century under RCP8.5. The largest changes in labor capacity were found in Northwest and North China, which were more than 40%, followed by East China (more than 10%) under RCP8.5.

The labor capacity of light work during the baseline period and the changes in the future are shown in Table 3. The labor capacity of light work was larger than that of heavy work for all regions of China. For the whole of China, the former (96%) was about 10% larger than the latter (85%) for both July and August in the baseline period. The difference was particularly significant in South China, where the labor capacity was less than 10% for heavy work while it was about 30% or more for light work. The decrease in labor capacity was a bit smaller for light work (about −3% to −4%) than for heavy work (−4% to −5%) under RCP2.6. Similar differences were found for RCP8.5 (i.e., the labor capacity of light work in China for July (August) would decrease by 4% (4.5%) and 15% (14.8%) during the 2021–2050 and 2071–2099 periods, respectively). In total, the labor capacity of light work would be larger than 90% in the near future and more than 80% in the late 21st century for the whole of China.

The largest labor capacity of light work during the baseline period was found in Northwest China and Northeast China, where the labor capacity was nearly 100% for July and August. The Tibetan Plateau had the next highest labor capacity at more than 96% for the two hottest months during the baseline period. The labor capacity in North China was also very high—more than 90% for July and 95% for August. It was about 30% (36%) and 50% (59%) for July (August) in East China and South China, respectively, which are the smallest regions. 

The decrease in the labor capacity of light work was largest in East and South China at −15% to −21% for July and −18% to −24% for August for the 2021–2050 period. The decrease was even larger for the period of 2071–2099 in these regions (i.e., −16% to 45% for July and −22% to −51% for August). The decreases in North China (about −35%) and Southwest China (about −28%) were also large for the late 21st century. The labor capacity could decrease slightly (−1% to −3% in Northeast China in the near future, but significantly decrease (−17% to −22%) in the late 21st century. The smallest changes in labor capacity were found in Northwest China and the Tibetan Plateau, which are decreasing by around 1% or less. It is noted that decreases in labor capacity in South and East China were significantly larger for light work than that for heavy work (see Table 2).

### 3.3. The Temporal Changes in the Labor Capacity in the Future

**Figure 7:**
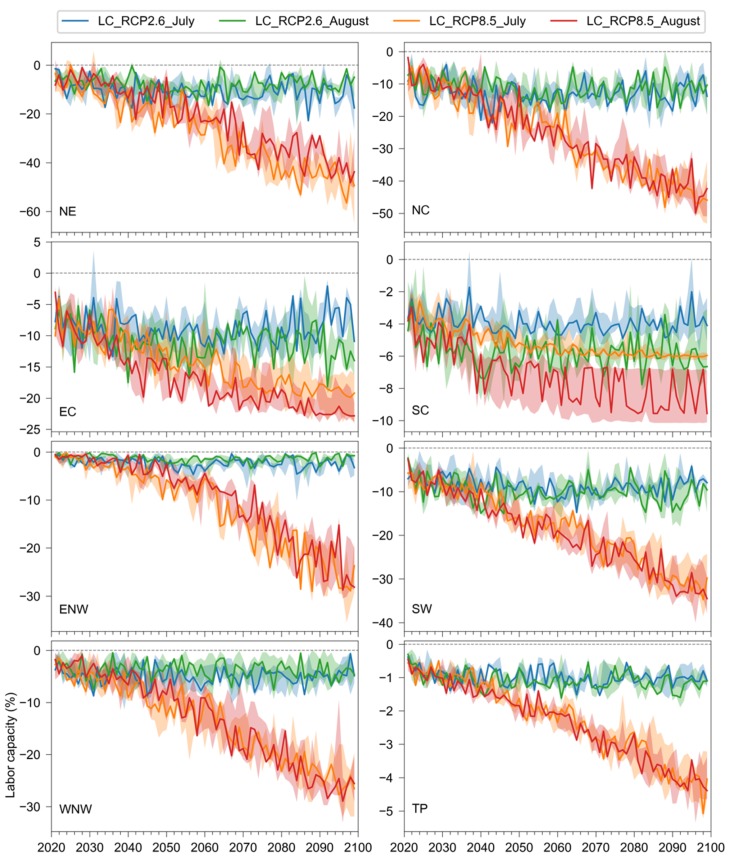
Changes in the annual labor capacity of heavy work for the eight regions in China from 2020 to 2099 compared to the baseline period (1980–2010) under RCP2.6 and RCP8.5. The lower and upper bounds of the shaded areas are the 25th and 75th percentiles from the multimodel ensemble.

Changes in the annual labor capacity of heavy work in the future are shown in Figure 7. Under RCP2.6, the changes in labor capacity were small in most years, with no trends found for the two months. However, under RCP8.5, the changes in annual labor capacity were estimated to significantly increase in the future. The downward trends of the changes in labor capacity were statistically significant in all regions under RCP8.5, while they were nonsignificant in most regions under RCP2.6. By the end of the century, the labor capacity would decrease by about 50% in Northeast and North China, and decrease by around 30% in Northwest and Southwest China. In East China, a labor capacity decrease of about 20% was found for the end of the century. The decreases in labor capacity were generally similar for July and August, but they were larger in August than in July for East and South China. It should be noted that uncertainty was considerable for all regions, especially for the data of August in South and East China, as indicated by the interquartile range (shaded areas) in Figure 7. 

**Figure 8:**
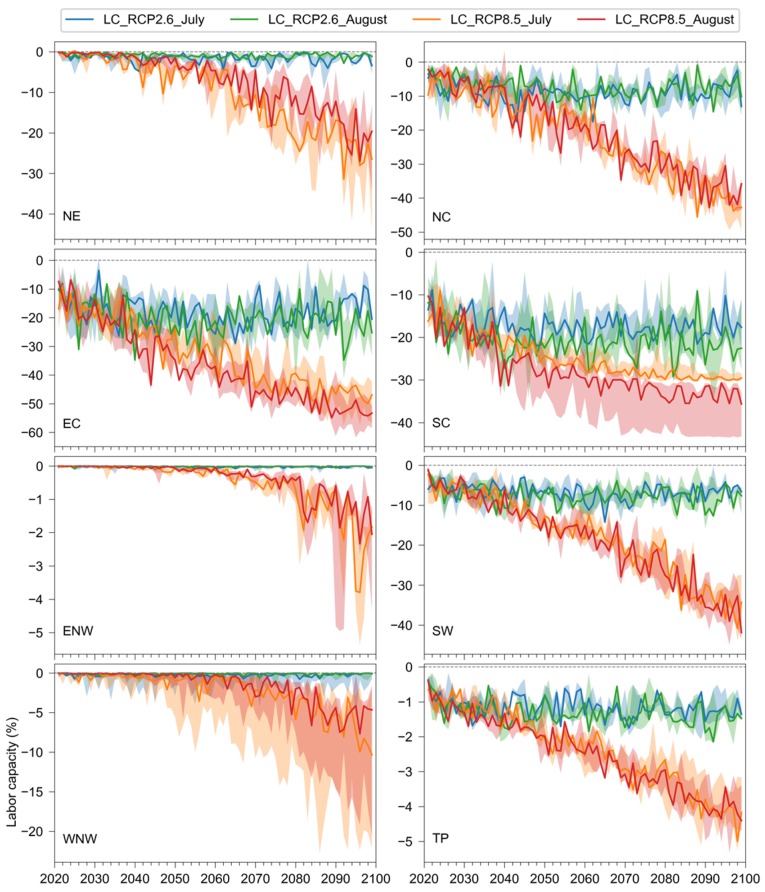
Changes in the annual labor capacity of light work for the eight regions in China from 2020 to 2099 compared to the baseline period (1980–2010) under RCP2.6 and RCP8.5. The lower and upper bounds of the shaded areas are the 25th and 75th percentiles from the multimodel ensemble.

Changes in the annual labor capacity of light work in the future are shown in Figure 8. Overall, the changes in the annual labor capacity of light work were smaller than that of heavy work (Figure 7) except for the South and East China. Under RCP2.6, the changes in labor capacity were small in most years and regions, showing no significant trends for the two months. However, under RCP8.5, the decreasing trends in annual labor capacity were more significant, particularly after 2050. By the end of the century, the labor capacity would decrease by more than 50% in East China, and decrease by around 40% in North, South, and Southwest China. The uncertainty of the changes in labor capacity were often larger under RCP2.6 than that under RCP8.5, especially in Northwest and South China. 

## 4. Discussion

Increasing heat stress would have significant socioeconomic implications in China during the two hottest months (i.e., July and August). By the end of the century, large decreases in labor capacity (particularly for heavy work) in the two months were found in most regions of China except for the Tibetan Plateau. In South China, the labor capacity almost approached zero in some years. In the future, the labor capacity of both heavy and light work would be very small (less than 10%) in most areas and less than 5% in some areas of South and East China. 

Most of the regions that showed large decreases in the labor capacity of heavy and light work, accommodate a large population and have a relatively developed economy (e.g., North China, East China, and South China). The labor capacity would be less than 50% in North China and less than 10% in East and South China. The decrease in labor capacity due to increased heat stress would reduce the productive working time and occupational labor for outdoor work. This may lower the production efficiency in the regions with high levels of outdoor jobs, and slow down regional economic development. In addition, occupational health with respect to heat stress will be a key issue in these regions in the future. The labor capacity remained high in northern and western China; however, the population is relatively small and economic growth is much slower there than eastern China. Thus, the reduction of labor capacity had fewer impacts in these regions, especially for light work.

Labor capacity is not directly related to the population working outdoors, but it can still represent the potential labor force that is appropriate for outdoor work in the future, since the latter can be inferred by scaling the total workforce with the labor capacity. On the other hand, the decrease in labor capacity will reduce productivity and increase labor costs. Occupational injuries may increase if people work outdoors for the same amount of time as they do presently. Diseases and injuries associated with heat stress may also increase among the ordinary population. This will cause significant damage and loss to human society and the economy [23,24].

It should be noted that considerable uncertainty might exist in the estimates of heat stress and labor capacities. This uncertainty may originate from GCMs (though the five GCMs are fairly representative [38]), the simplified equation of the WBGT [7,39], and also the empirical relationships between labor capacity and the WBGT [29]. We estimated the relationships between labor capacity and WBGT for only heavy and light work but not moderate work, aiming to give a range of changes in labor capacity caused by heat stress. Further investigations may be needed for more details on the changes with respect to different occupations and occupational risks/hazards. 

The increase in WBGT partly results from higher air temperature, and thus spatial patterns of the WBGT are often similar to those of high temperature [7]. However, the changes in humidity would also affect the heat stress indicated by the WBGT. Therefore, although July is often the hottest month with respect to dry air temperature, the WBGT can be higher in August than in July, especially in eastern China. Thus, relative humidity cannot be ignored in mitigating heat-related damages under global warming.

## 5. Conclusions

In this study, we examined the changes in heat stress based on the wet-bulb globe temperature (WBGT), and estimated the changes in labor capacity of heavy and light work caused by heat stress during the hottest two months (July and August) in China in the future under RCP2.6 and RCP8.5 scenarios. Results showed that the WBGT would increase by more than 1 °C and by 3–5 °C in those two months during the 2021–2050 and 2071–2099 periods, respectively. This indicates significantly intensified heat stress in China. Large decreases (more than 40%) in labor capacity of heavy work due to increased WBGT were found for many areas of China in the future, especially at the end of the century under RCP8.5. In South and East China, labor capacity of light work would also experience a significant decrease (by 40% to 50%) under the high emission scenario. The large decreases in labor capacity generally would occur in the regions with high population densities and developed economies. This could have significant implications on regional economic development as well as on occupational health associated with heat stress.

## Figures and Tables

**Table 1:** Threshold limit values of the wet-bulb globe temperature (WBGT) for different manual works.

Labor Capacity (%)	Workload			
	I	II	III	IV
100	30	28	26	25
75	31	29	28	26
50	32	30	29	28
25	33	32	31	30

**Note:** Workload I indicates light manual work or the movement of legs (e.g., typewriting, sewing, operating equipment, etc.); Workload II indicates moderate manual work with continuous movement of the hands, arms, legs, and body (e.g., sawing, forging, weeding whitewashing, etc.); Workload III indicates relatively heavy manual work like lifting, shoveling, digging, etc.); Workload IV indicates very heavy manual work like digging and lifting at high intensity, activities that involve a high rhythm of the heart.

**Table 2:** Labor capacity of heavy works during the baseline period and their changes in the 2021–2050 (2035) and 2071–2099 (2085) periods under RCP2.6 and RCP8.5. Changes in labor capacity are calculated as the differences between the future periods and the baseline period.

**RCP26_July**	**Baseline**	**2035**	**2085**	**RCP85_July**	**Baseline**	**2035**	**2085**
NE	85.39	−6.96	−9.49	NE	85.39	−12.10	−42.07
NC	67.43	−13.10	−14.43	NC	67.43	−15.28	−40.20
EC	20.10	−7.39	−7.18	EC	20.10	−10.76	−18.29
SC	6.00	−3.39	−3.59	SC	6.00	−4.27	−5.92
ENW	99.11	−2.39	−2.48	ENW	99.11	−3.49	−22.01
SW	64.69	−7.69	−9.36	SW	64.69	−11.26	−29.06
WNW	95.03	−4.10	−4.36	WNW	95.03	−6.57	−22.39
TP	93.14	−0.86	−0.94	TP	93.14	−1.05	−3.64
CN	85.21	−4.00	−4.57	CN	85.21	−5.52	−17.19
**RCP26_August**	**Baseline**	**2035**	**2085**	**RCP85_August**	**Baseline**	**2035**	**2085**
NE	91.01	−6.91	−9.40	NE	91.01	−8.78	−34.91
NC	72.88	−10.76	−11.87	NC	72.88	−13.73	−40.04
EC	24.12	−10.89	−12.10	EC	24.12	−11.95	−21.41
SC	9.82	−5.45	−5.30	SC	9.82	−5.04	−8.21
ENW	99.47	−1.30	−1.87	ENW	99.47	−2.30	−19.85
SW	66.26	−9.52	−11.13	SW	66.26	−10.42	−27.88
WNW	97.63	−3.34	−3.86	WNW	97.63	−5.13	−23.36
TP	93.10	−1.03	−1.29	TP	93.10	−1.11	−3.78
CN	85.67	−4.23	−4.57	CN	85.67	−5.55	−15.82

**Table 3:** Labor capacity of light works in the baseline period and the changes in the 2021–2050 (2035) and 2071–2099 (2085) periods under RCP2.6 and RCP8.5. Changes in labor capacity are calculated as the differences between the future periods and the baseline period.

**RCP26_July**	**Baseline**	**2035**	**2085**	**RCP85_July**	**Baseline**	**2035**	**2085**
NE	99.47	−1.46	−1.30	NE	99.47	−2.79	−22.14
NC	92.44	−7.99	−10.13	NC	92.44	−10.26	−35.67
EC	51.60	−15.42	−16.13	EC	51.60	−21.28	−44.65
SC	29.72	−15.18	−16.98	SC	29.72	−18.81	−29.36
ENW	99.99	−0.05	−0.04	ENW	99.99	−0.07	−1.13
SW	88.24	−5.24	−6.59	SW	88.24	−8.22	−28.39
WNW	99.94	−0.17	−0.21	WNW	99.94	−0.39	−4.58
TP	96.14	−1.03	−1.10	TP	96.14	−1.18	−3.66
CN	95.64	−2.84	−3.32	CN	95.64	−4.03	−15.05
**RCP26_August**	**Baseline**	**2035**	**2085**	**RCP85_August**	**Baseline**	**2035**	**2085**
NE	99.77	−0.85	−1.23	NE	99.77	−1.78	−17.07
NC	95.36	−5.80	−8.48	NC	95.36	−9.13	−33.87
EC	59.38	−18.11	−22.53	EC	59.38	−24.07	−51.19
SC	36.71	−21.57	−21.88	SC	36.71	−21.19	−30.48
ENW	100.00	−0.02	−0.02	ENW	100.00	−0.03	−1.05
SW	89.18	−7.63	−8.07	SW	89.18	−8.39	−27.49
WNW	99.98	−0.09	−0.09	WNW	99.98	−0.14	−3.47
TP	96.09	−1.41	−.56	TP	96.09	−1.32	−3.78
CN	95.78	−3.24	−3.67	CN	95.78	−4.46	−14.82
